# Association between biomarkers of bone health and osteosarcopenia among Iranian older people: The Bushehr Elderly Health (BEH) program

**DOI:** 10.1186/s12877-021-02608-w

**Published:** 2021-11-19

**Authors:** Maryam Fathi, Ramin Heshmat, Mehdi Ebrahimi, Ahmad Salimzadeh, Afshin Ostovar, Ali Fathi, Farideh Razi, Iraj Nabipour, Maryam Moghaddassi, Gita Shafiee

**Affiliations:** 1 Chronic Diseases Research Center, Endocrinology and Metabolism Population Sciences Institute, Tehran University of Medical Sciences, Tehran, Iran; 2 Department of Internal Medicine, Faculty of Medicine, Sina Hospital, Tehran University of Medical Sciences, Tehran, Iran; 3 Rheumatology Research Center, Sina Hospital, Tehran University of Medical Sciences, Tehran, Iran; 4 Osteoporosis Research Center, Endocrinology and Metabolism Clinical Sciences Institute, Tehran University of Medical Sciences, Tehran, Iran; 5 Isfahan university of medical science, Isfahan, Iran; 6 Endocrinology and Metabolism Research Center, Endocrinology and Metabolism Clinical Sciences Institute, Tehran University of Medical Sciences, NO 10, Jalale-Al-Ahmad Ave, Chamran Highway, Tehran, Iran; 7 The Persian Gulf Tropical Medicine Research Center, Bushehr University of Medical Sciences, Bushehr, Iran

**Keywords:** Osteosarcopenia, Bone turnover markers, Older people

## Abstract

**Background:**

Osteosarcopenia is referred to as co-incidence of osteoporosis/osteopenia and sarcopenia which is defined as a geriatric syndrome with a significant prevalence that increases morbidity and mortality. There are some relevant factors that can show an increased risk of incidence of osteosarcopenia.

**Aim:**

We aimed to consider the association of bone turnover markers such as Osteocalcin (OC), C-terminal cross-linked telopeptide (CTX), Tartrate Resistant acid Phosphatase (TRAP), Bone Alkaline Phosphatase (BALP) and also other factors like vitamin D, calcium, phosphorous, and ALP with osteosarcopenia in elderly.

**Methods:**

We carried out a cross-sectional study on a random sample including 400 elder participants of Bushehr Elderly Health (BEH) study, in Iran. Osteopenia/ osteoporosis was defined as a T-score ≤ -1.0 standard deviation below the mean values of a young healthy adult. We defined sarcopenia as low muscle strength (handgrip strength<26 kg for men and <18 kg for women) with reduced skeletal muscle mass [Skeletal muscle index (SMI) < 7.0 kg/m^2^ for male and <5.4 kg/m^2^ for female]. Osteosarcopenia was considered as the presence of both osteopenia/osteoporosis and sarcopenia. We estimated the age-standardized prevalence of osteosarcopenia for men and women, separately. We used multivariable logistic regression to address the factors associated with osteosarcopenia.

**Results:**

The results showed that there was a statistically significant difference in OC), CTX, TRAP were between the osteosarcopenia (-) and osteosarcopenia (+) groups. No statistically significant difference was observed in BALP, vitamin D, calcium, phosphorous, and ALP between the compared groups.

In the multivariable logistic regression model, OC and CTX were associated with increased likelihood of osteosarcopenia [adjusted OR= 1.023(1.002-1.045 for OC, 4.363(1.389-15.474 for CTX)]. Furthermore, TRAP increases the odds of osteosarcopenia in crude model [OR= 1.333 (1.070- 1.660)].

**Conclusions:**

We observed the association between bone turnover markers particularly OC, CTX and osteosarcopenia. Given the rapid growth of the aging population, we should focus on geriatric diseases such as musculoskeletal disorders. Bone turnover markers maybe improve the early diagnosis, screening and assess the response to therapies in people with osteosarcopenia.

**Supplementary Information:**

The online version contains supplementary material available at 10.1186/s12877-021-02608-w.

## Introduction

Osteopenia and sarcopenia are two important musculoskeletal disorders with adverse outcomes in older people. The scenario could be more devastating when these conditions are present at the same time in the same patient. Osteosarcopenia is recently defined medical terms that refer to the co-incidence of both osteopenia and sarcopenia [1]. It is associated with higher fracture, morbidity, length of stay at the hospital, and mortality in elder people and consequently could lead to increased burden of disease [2, 3]. In such phenomenon, normal function both of muscle and bone tissues is interrupted due to loss of bone density and decrease in muscle mass and strength [2, 4, 5]. As the population is getting older, the prevalence of osteosarcopenia is increasing [3, 6]. Musculoskeletal disorders are highly prevalent in developed countries [3, 7] and the same patterns are expected for developing countries like Iran that are faced with health transition and population aging in the recent area [8–10]. Musculoskeletal disorders are also important in terms of economic issues as they impose a large amount of direct and indirect costs on people, health systems and societies [11].

Several risk factors have already been addressed for osteoporosis and sarcopenia. According to previous studies age, female gender, BMI, tobacco smoking, alcohol consumption, and some drugs are associated with such musculoskeletal disorders [12–14].

Assessment of musculoskeletal condition is possible by several preclinical methods which one of them is measuring the level of bone turnover markers from serum or urine sampling. The result of these laboratory data gets ready faster and more convenient compared to other predictive bone health preclinical methods such as Bone mineral densitometry (BMD). The biomarkers are available for the assessment of bone turnover condition, including enzymes and non-enzymatic peptides which are derived from the cellular and non-cellular compartments of bone [15].

Bone turn over markers are categorized into two groups based on the metabolic phase during which they are listed below:

1. Bone formation markers: Osteocalcin (OC), Bone Alkaline Phosphatase (BALP).
2. Bone resorption markers.: C-terminal telopeptide (CTX), Tartrate Resistant acid Phosphatase (TRAP)..

Bone biomarkers are novel tools that assess the dynamics of bone remodeling with respect to bone formation and resorption [16]. The association between bone turnover markers and osteoporosis are studied before in recent decades [16, 17]. The high bone turnover markers could predict the risk of osteoporotic fractures in postmenopausal women. These markers not only detect bone remodeling and diagnosis but also provide information on the therapeutic monitoring of osteoporosis [16, 18]. Furthermore, osteoporosis and sarcopenia are two important diseases with overlapping risk factors and pathogenesis. Common biochemical pathways, mechanical and endocrine factors have been identified that affect both muscle and bone units [19]. Previous studies found that a relationship between bone markers and sarcopenia [20]. It seems that these factors are necessary to maintain muscle mass with promoting protein synthesis [21].

Therefore, when occurring together osteoporosis and sarcopenia, there are intensive and complex interactions, both mechanically and biochemically [22].

In the present study, we aimed to evaluate the association between laboratory and bone turnover biomarkers with osteosarcopenia in our study population. Bone turnover markers maybe improve the early diagnosis, and screening in people with osteosarcopenia.

## Materials and methods

We carried out a cross-sectional study on a random sample of the second stage of BEH program. The BEH (Bushehr Elderly Health) program is a population-based prospective cohort study being performed in Bushehr, a southern province in Iran In brief, overall 3000 persons aged ≥60 years were recruited using a multistage, stratified cluster sampling method [23]. The second stage of the BEH program [24], 2426 participants were included to investigate on musculoskeletal health. In current study, a random sample from stage II of BEH study was selected to assess the bone turnover marker level and some other biochemical laboratory tests such as Vitamin D, calcium, and phosphor in the blood samples.

Exclusion criteria were intake of immunosuppressive drugs, history of hyper-parathyroid, history of cancer, chronic kidney and liver disease, sarcoidosis. Therefore, 397 individuals aged ≥60 years without missing data of bone markers and free from anti-osteoporotic drugs, calcium ,and vitamin D supplementation since at least 6 months were included in this study.

The study protocol was reviewed and approved by the Research Ethics Committee of both Bushehr University of Medical Sciences and Endocrinology and Metabolism Research Institute and also ethics committee of Tehran University of Medical Sciences. All methods in thisstudy were performed in accordance with the relevant guidelines and regulations. The study participants completed written informed consent before the study.

Data were collected through the comprehensive questionnaires including sociodemographic characteristics, general health, medical history, and lifestyle data during an interview that was performed by a trained interviewers. A fixed stadiometer and a digital scale were used for the measurements of height and weight, respectively. Waist circumference (WC) was measured at a point midway between the iliac crest and the lowest rib in standing position. Body mass index (BMI) was calculated by the formula weight (kg) / [height (m)]^2^. The physical activity level was evaluated by a standard questionnaire-based on metabolic equivalent (MET) levels [25]. The activities were included in the physical activity scale, organized in nine different MET levels ranging from sleep/rest (0.9 METs) to high-intensity physical activities (6 METs). The time of each activity multiped of MET level in 24 h named physical level of each individual. According to physical activity level, four lifestyle categories are defined (sedentary: 1-1.39, low active: 1.4-1.59, active: 1.6-1.89, very active: 1.9-2.5). We divided the study population into two categories; low physical activity (sedentary and low active) and high physical activity (active and very active).Blood pressure (BP) was measured twice in a seated position after 15 min rest a standard mercury sphygmomanometer.

Dual x-ray absorptiometry (DXA, Discovery WI, HologicInc, USA) was used to measure bone mineral density, fat mass, and muscle mass. In BMD assessment at L1–L4 level, first requirement is correct positioning of patient. The spinous process should be centered in straight midline and should include part of the sacrum (ilium) and part of a vertebra with ribs (usually T12).In BMD assessment at femoral neck, lesser trochanter should be just visible.

To evaluate skeletal muscle mass, we used total body scans using DXA.we calculated appendicular skeletal muscle mass (ASM) as the sum of lean mass from four limbs. A skeletal muscle mass index (SMI) was defined as ASM divided by squared height.

Muscle strength was measured by handgrip strength, using a digital dynamometer. The participant seated, elbow at side and 90^0^ and the hand in a neutral position. The measurement was carried out three times for each hand and maximum grip strength was calculated by taking the highest measurement from both hands [26]. Usual walking speed (m/s) on a 15 feet (4.57-meter) course measured manually with a stopwatch to measure gait timing [27, 28]. A single cut-off speed ≤0.8 m/s was called low performance for both genders [29].

### Definition of terms

Osteosarcopenia (+) was the main interesting outcome of the current study that is defined as a new syndrome contain both osteoporosis/osteopenia and sarcopenia at the same time. According to WHO standard criteria [30], bone mineral density (BMD) lower than 1 SD below the reference mean (T-Score ≤ -1) in either the femoral neck, lumbar spine or total hip were categorized as osteopenia/ osteoporosis [31]. Diagnosis of sarcopenia was based on low muscle strength and low muscle mass according to the European Working Group on Sarcopenia in Older People (EWGSOP-2) with Iranian cutoff point. Low muscle mass was defined as the SMI lower than 7.0 kg/m^2^ for male and 5.4 kg/m^2^ for female, low muscle strength was lower than 26 kg for male and 18 kg for female, and low physical performance was lower than 0.8 m/s for both genders [10, 29, 32]. People without osteoporosis/osteopenia and sarcopenia at the same time named Osteosarcopenia (-).

Current smokers was defined as participants who who smokes at least one cigarette per day or uses a hookah or pipe once daily at the time of evaluation. Diabetes mellitus type 2 was defined as the amount of fasting blood glucose above 126 mg/dl or taking any anti-diabetic medication. Hypertension was defined as SBP ≥ 140 mmHg or DBP ≥ 90 mmHg or taking any anti-hypertensive medications. Hypercholesterolemia was defined as TC ≥200 mg/dl, low HDL-C as < 40 mg/dl for men and < 50 mg/dl for women, and hypertriglyceridemia as TG ≥150 mg/dl [33].

High-fat mass is described as total body fat percent > 30 % for males and > 40 % for females measuring by body composition analyzer [34].

### Biochemical measurements

Patients’ lipid profile and blood glucose were measured by assessing venous samples, drawn after overnight fasting. Using the enzymatic colorimetric method with cholesterol esterase and cholesterol oxidase, total cholesterol (TC) was determined. Details for the measurements of fasting plasma glucose (FPG), high-density lipoprotein (HDL-C), low-density lipoprotein cholesterol (LDL-C) and triglyceride (TG) were reported elsewhere [24]. In this study, We collected data on serum bone biomarkers including Osteocalcin (OC), Bone Alkaline Phosphatase (BALP), C-terminal cross-linked telopeptide (CTX), Tartrate Resistant acid Phosphatase (TRAP), and also, vitamin D, calcium, phosphor and Alkaline Phosphatase (ALP).

OC and CTX were measured by electrochemiluminescence immunoassay method (Roche Diagnostics GmbH). TRAP, BALP, and 25-Hydroxy Vitamin D levels were measured by ELISA method (Immunodiagnostic systems, UK). Calcium (arsenazo), Phosphorous (Phosphophomolybdate) and ALP (DGKC) were measured by commercial kits (parsazmoon, Tehran, Iran) (Supplement Table 1).

**Table 1:** Study participants demographic and clinical characteristics

	Total (n=397)	Osteosarcopemnia(-) (n=321)	Osteosarcopenia(+) (n=76)	P-value
Sex, n (%)				
Women	211 (53.1)	169 (52.6)	42 (55.3)	0.681
Men	186 (46.9)	152 (47.4)	34 (44.7)	
Age (Years)	69.2 ±6.3	68.3 ±5.7	72.9 ±7.3	<0.001
Weight (Kg)	69.5±12.9	72.2±12.1	58.2±9.9	<0.001
Height (Cm)	158.4±9.1	159.0±9.1	155.9±8.8	0.006
Waist circumference (Cm)	99.1±11.2	100.9±10.7	91.3±9.4	<0.001
Body mass index (Kg/m^2^)	27.7 ±4.7	28.6±4.7	23.8 ±2.8	<0.001
Education (Years)	4.9 ±5.1	5.4 ±5.1	2.7 ±4.4	<0.001
Current Smoking, n (%)	71 (17.9)	57(17.8)	14(18.4)	0.901
Physical activity, n (%)	87 (21.9)	79(24.6)	8 (10.5)	0.008
High fat mass, n (%)	280 (71.2)	234 (73.8)	46 (60.5)	0.021
Diabetes mellitus, n (%)	105 (26.4 %)	79 (24.6)	26 (34.7)	0.076
Systolic blood pressure (mmHg)	140.5±18.3	141.4±18.1	136.9±18.8	0.053
Diastolic blood pressure(mmHg)	81.4±8.4	82.0±8.4	78.8±7.9	0.003

Data are presented as mean (standard deviation), number (percent)

### Statistical analysis

Quantitative data were described as mean and standard deviation (SD) for normally distributed data, based on the Shapiro-Wilk test, while non-normal variables were presented as median (interquartile range: P25-P75). Categorical data were explained by numbers and percentage. The osteosarcopenic and non-osteosarcopenic populations were compared for the baseline characteristics using Pearson’s Chi-square test for the categorical, and independent sample t-test for continues variables.We used multivariable logistic regression to examine the association between biomarkers and osteosarcopenia. Adjusted for confounding variables. Adjustment was done for the variables that showed a p-value of 0.2 or lower in univariable analysis.

Data were analyzed using the Stata 14 software (StataCorp. 2015. Stata Statistical Software: Release 14. College Station, TX: StataCorp LP) and P≤0.05 was considered as statistically significant in all tests.

## Results

Among 397 participants, 19.1 % had osteosarcopenia. Overall, 211 study participants were female and no statistically significant difference was observed regarding gender distribution between the compared groups. The total average age of study participants was 69.2±6.3 years and the osteosarcopenia (+) group (72.9±7.3 years) was significantly older than the osteosarcopenia (-) group (68.3±5.7 years) (P-value<0.001). Education level in the osteosarcopenia (-) group was considerably higher than osteosarcopenic subjects (P-value<0.001). Current tobacco smoking was reported by 71 participants (17.9 %) and the proportion of diabetes was higher in osteosarcopenic people than people without osteosarcopenia. We compared BMI, physical activity, waist circumference and body fat mass between osteosarcopenia (-) and osteosarcopenia (+) groups, and all the investigated characteristics were pretty higher in the osteosarcopenia (-) group. According to Table 1 mean BMI (28.6 vs. 23.8) and proportion of high body fat mass (73.8 % vs. 60.5 %) were drastically higher in the osteosarcopenia (-) group in comparison to the patients with Osteosarcopenia (P-value<0.05). Besides, we highlighted that osteosarcopenia (-) group was physically more active than osteosarcopenic patients (P-value= 0.008) (Table 1).

In Table 2 we compared bone biomarkers between osteosarcopenia (-) and osteosarcopenia (+) participants. According to Table 2, the median of OC was 21.4 ng/ml for the osteosarcopenia (-) group, while it was 24.0 ng/ml in osteosarcopenia (+) group (P-value=0.043). Moreover, the reported values of CTX for participates without Osteosarcopenia (median= 0.395 ng/ml) was significantly lower than osteosarcopenia (+) patients (median = 0.465 ng/ml) (P-value= 0.014). We also observed a statistically significant difference between compared groups regarding TRAP where the median of TRAP in osteosarcopenia (-) and osteosarcopenia (+) groups were 3.30 U/L and 3.65 U/L, respectively (P-value=0.001). No statistically significant difference was observed in BALP, vitamin D, calcium, phosphorous, and ALP between the compared groups (P-value>0.05) (Table 2).

**Table 2:** Comparison of biomarkers between normal and Osteosarcopenia groups

Bone marker	Osteosarcopenia(-)	Osteosarcopenia(+)	P-value
Osteocalcin, ng/ml	21.40 (16.90-28.35)	24.05 (18.32-31.60)	0.043
C-terminal cross-linked telopeptides (CTX), Pg/ml	0.395 (0.29-0.56)	0.465(0.340-0.598	0.014
Bone alkaline phosphatase(BALP), U/L	15.85 (12.5-20.15)	16.29 (13.20-20.90)	0.283
Tartrate-resistant acid phosphatase (TRAP), U/L	3.30 (2.70-4.00)	3.65 (3.20-4.30)	0.001
Vitamin D,nmol/l	42.20 (24.95-62.05)	40.95 (27.78-64.83)	0.653
Calcium, mg/dl	9.30 (9.00-9.60)	9.30 (9.10-9.50)	0.702
Phosphorus, mg/dl	4.01 (3.61-4.34)	4.16 (3.70-4.58)	0.111
Alkaline phosphatase, U/L	206.5 (181.0-240.0)	200.0 (169.0-237.0)	0.647

Data are presented as median(Interquartile range)

The association between osteosarcopenia and bone biomarkers was also investigated adjusted for potential variables including age, sex, BMI, high fat mass, education level, physical activity, and diabetes status using the multivariable logistic regression models. The results showed that OC and CTX were associated with increased likelihood of osteosarcopenia (OC: adjusted OR= 1.023, 95 % CI= 1.002-1.045; CTX: adjusted OR= 4.363, 95 % CI= 1.389- 15.474). Furthermore, crude model showed that TRAP increases the odds of osteosarcopenia (crude OR= 1.333, 95 % CI= 1.070- 1.660), and this association after adjusted for other factors was disappeared (Table 3).

**Table 3:** Relationship between biomarkers and osteosarcopena in different models

Exposure	Model 1 (OR (95 % CI)	Model 2 (OR (95 % CI)
Osteocalcin	1.019(1.003-1.035)	1.023(1.002-1.045)
C-terminal cross-linked telopeptides (CTX)	3.552(1.472-8.573)	4.363(1.389-15.474)
Bone alkaline phosphatase(BALP)	1.009(0.986-1.033)	1.017(0.991-1.044)
Tartrate-resistant acid phosphatase (TRAP)	1.333(1.070-1.660)	1.170(0.870-1.574)
Vitamin D	1.002(0.996-1.008)	1.001(0.994-1.008)
Calcium	0.863(0.539-1.383)	1.038(0.584-1.846)
Phosphorus	1.376(0.878-2.158)	1.075(0.594-1.947)
Alkaline phosphatase	0.999(0.996-1.003)	1.001(0.997-1.005)

Model; No adjustment

Model 2; adjusted for age, sex, Body mass index, diabetes, physical activity, education, High fat mass

## Discussion

In the current cross-sectional study, we evaluated the relationships between bone markers and osteosarcopenia. We found higher serum of OC, CTX, and TRAP in participants with osteosarcoepenia compared with people without osteosarcopenia. Also, these factors were positively associated with osteosarcopenia in crude models and OC and CTX remained even after adjusting for other confounding factors.

Some studies showed that there is the interaction between muscle and bone in health and disease [17, 35]. These two organs are connected through biochemical pathways, mechanical and endocrine factors. For example, insulin resistance, decreased glycogen synthesis and mitochondrial dysfunction cause muscle loss in diabetes states. Oxidative stress, chronic hyperglycemia and advanced glycation end products (AGEs) also may decrease bone formation and suppress differentiation of myogenic genes [35, 36]. It suggested a common mechanism via bone- muscle interaction. In this regard, the results of our study also showed that individuals with osteosarcopenia have more endocrine dysfunction such as diabetes.

Bone markers represent the molecules directly connected to both the structure and function of bone tissue. Bone remodeling is composed of some bone formation and bone resorption markers. These factors indirectly influence muscle [17]. Of those, osteocalcin with the main role in skeletal remodeling regulates β-cells and insulin secretion in muscle and therefore is effective on muscle mass and strength [35]. Also, OC directly promotes protein synthesis in myotubes and thus it can responsible for muscle maintenance during aging [21].

In many studies, the levels of osteocalcin have been different between osteoporotic and non- osteoporotic people and serum OC may be useful for the assessment of osteoporosis and the prediction of the fracture risk in older people [17, 37]. A recent study found that the relationship between OC and skeletal muscle mass and muscle function in postmenopausal women [20]. So, favorable effects of OC on the interaction between bone and muscle can be suggested in osteosarcopenic people. Our findings also showed that an association between OC and osteosarcopenia according to the multivariable logistic regression. Drey et al. demonstrated OC was significantly increased in osteosarcopenic people and their results were similar to our findings in this regard [22]. The diverse association between OC and bone density has already been shown in previous studies and it could be considered as the underlying cause of the increased risk of osteosarcopenia in those with higher OC [38]. Muraca et al. have highlighted the role of extracellular vesicles (EVs) in causal pathway of OC and Osteosarcopenia [39]. EVs are known as one essential factor for intercellular communication and play a major role in signaling and expression of couple molecules including lipids, proteins, and nucleic acids and any change in such agents will cause gene expression and their function. As a result the change in EVs is associated with change in OC density and consequently affect the incidence of osteosarcopenia [39]. Several other studies have reported OC as a predictive factor of fracture and osteosarcopenia in elder people [40, 41]. However, in a couple of other studies, no association between OC and osteosarcopenia has been reported that was in contrast with our findings [13]. In this study, only postmenopausal women were recruited that was completely different from the investigated population in the current study and different findings might be due to different study population.

Another bone turnover factor is CTX as a bone resorption biomarker that is released during collagen degradation [17]. This marker increases with age and menopause and serum levels may predict bone loss and fracture risk. Also, CTX is a specific and sensitive bone resorption marker that rapidly shows the response to treatment in postmenopausal osteoporotic women [42].

Moreover, we observed an increased likelihood of osteosarcopenia in elders with a higher level of CTX similar to other study in osteosarcopenic people [22].

However, some studies have shown the relationship between other bone turnover biomarkers such as TRAP, BALP with osteoporosis, relatively few studies have examined the association these markers with skeletal muscle and also combined indices of body composition [17, 19]. Our results showed that the TRAP was significantly higher in osteosarcopenic people and it was associated with risk of osteosarcopenia in the crude model. However, after adjustment for confounding variables this association disappeared.

Although in this study, we showed that the rate of many bone markers is higher in osteosarcopenic individuals, we performed an additional analysis to determine whether this association is due to osteopenia or osteopenia with sarcopenia (osteosarcopenia). In this analysis, individuals were divided into three groups include; individuals with osteopenia/ osteoporosis (+), osteosarcopenia (+), and people without osteopenia/ osteosarcopenia (control group). The results showed that the medians of bone markers; OC, CTX, and TRAP were significantly higher in people with osteosarcoma than in people with osteopenia. This suggests that the presence of osteopenia + sarcopenia (osteosarcopenia) beyond osteopenia alone is involved in the association of markers with this phenomenon (Supplement Table 2).

Other factors influencing muscle and bone metabolism include deficiencies in calcium, and vitamin D, which affect the quality of these tissues. Because of bone is the largest source of calcium and its intake can prevent from osteoporosis [43]. Also, calcium influences on neuromuscular function and therefore it can reduce sarcopenia [44]. The role of vitamin D in protection of bone and muscle is well established. Previous studies showed that people with low vitamin D have low muscle mass and low bone mass and it seems that the deletion of the vitamin D receptor in bone and muscle is as a possible mechanism for reducing of function of both tissue [45, 46].We found no association between vitamin D level and risk of osteosarcopenia, whereas in two previous studies vitamin D has been demonstrated as one of contributing factor in the etiology of osteosarcopenia. According to Kim et al. patients with osteosarcopenia was associated with increased vitamin D level, while we did not found such association [47]. Kim et al., have performed their study on obese people and their sample was different from our study participants in this regard. Moreover, vitamin D deficiency is considered public health challenge and most the Iranian people have the level of vitamin D deficiency. According to one study in Iranian adults, the prevalence of mild, moderate, and severe vitamin D deficiency was 19.6 %, 23.9 %, and 26.9 %, respectively that shows our sample was homogenous in terms of vitamin D deficiency [48]. In addition, results of a carried out systematic review have shown a high level of heterogenic in results of previous studies regarding the association between vitamin D and osteosarcopenia and it is still regarded as a controversial issue [49].

We carried out our study on a community-based subject’s recruited from a population-based health program that is a better representative of the Iranian elder population. Moreover, the current study is one the least attempts to investigate the association of bone biomarkers with osteosarcopenia etiology. However, the current study is not free from limitations, and our findings must be interpreted in the context of our limitations. We had a relatively low sample size and limited statistical power that prohibited us to perform subgroup analysis and could reduce the generalizability of our findings. Also, we did not have the data of Pro-collagen type 1 N-terminal Propeptide (P1NP) as the most sensitive biomarker to measure bone formation rate in osteoporosis. Moreover, we were not able to assess temporality due to cross-sectional nature of our study.

## Conclusions

We found a valuable association between a high level of OC and CTX and incidence of osteosarcopenia which these bone turnover markers can easily check in serum sampling and can have an important role in estimating the risk of osteosarcopenia incidence. Given the rapid growth of the aging population, we should focus on geriatric diseases such as musculoskeletal disorders. Bone turnover markers maybe improve the early diagnosis, screening and assess the response to therapies in people with osteosarcopenia.

## Supplementary Information

**Additional file 1.**

## Data Availability

The data that support the findings of this study are available from the principal investigator (Prof. Iraj Nabipour, Email: inabipour@gmail.com) of Bushehr Elderly Health (BEH) program but restrictions apply to the availability of these data, which were used under license for the current study, and so are not publicly available.

## References

[1] Binkley N, Buehring B (2009). Beyond FRAX: it’s time to consider” sarco-osteopenia". J Clin Densitometry.

[2] Edwards M, Dennison E, Sayer AA, Fielding R, Cooper C (2015). Osteoporosis and sarcopenia in older age. Bone.

[3] Paintin J, Cooper C, Dennison E (2018). Osteosarcopenia. Br J Hosp Med (Lond).

[4] Fuggle N, Shaw S, Dennison E, Cooper C (2017). Sarcopenia. Best Practice Res Clin Rheumatol.

[5] Huo YR, Suriyaarachchi P, Gomez F, Curcio CL, Boersma D, Muir SW, Montero-Odasso M, Gunawardene P, Demontiero O, Duque G (2015). Phenotype of osteosarcopenia in older individuals with a history of falling. J Am Med Directors Assoc.

[6] Fahimfar N, Zahedi Tajrishi F, Gharibzadeh S, Shafiee G, Tanha K, Heshmat R, Nabipour I, Raeisi A, Jalili A, Larijani B (2020). Prevalence of Osteosarcopenia and Its Association with Cardiovascular Risk Factors in Iranian Older People: Bushehr Elderly Health (BEH) Program. Calcif Tissue Int.

[7] Shafiee G, Keshtkar A, Soltani A, Ahadi Z, Larijani B, Heshmat R (2017). Prevalence of sarcopenia in the world: a systematic review and meta- analysis of general population studies. J Diabetes Metab Disord.

[8] Shafiee G, Heshmat R, Ostovar A, Nabipour I, Larijani B (2019). Sarcopenia disease in Iran: an overview. J Diabetes Metabolic Disorders.

[9] Fahimfar N, Noorali S, Yousefi S, Gharibzadeh S, Shafiee G, Panahi N, Sanjari M, Heshmat R, Sharifi F, Mehrdad N (2021). Prevalence of osteoporosis among the elderly population of Iran. Arch Osteoporos.

[10] Shafiee G, Heshmat R, Ostovar A, Khatami F, Fahimfar N, Arzaghi SM, Gharibzadeh S, Hanaei S, Nabipour I, Larijani B (2020). Comparison of EWGSOP-1and EWGSOP-2 diagnostic criteria on prevalence of and risk factors for sarcopenia among Iranian older people: the Bushehr Elderly Health (BEH) program. J Diabetes Metab Disord.

[11] Hernlund E, Svedbom A, Ivergård M, Compston J, Cooper C, Stenmark J, McCloskey EV, Jönsson B, Kanis JA (2013). Osteoporosis in the European Union: medical management, epidemiology and economic burden. Archives Osteoporosis.

[12] Kim J, Lee Y, Kye S, Chung YS, Lee O (2017). Association of serum vitamin D with osteosarcopenic obesity: Korea National Health and Nutrition Examination Survey 2008–2010. J Cachexia Sarcopenia Muscle.

[13] Ardawi MSM, Rouzi AA, Al-Sibiani SA, Al‐Senani NS, Qari MH, Mousa SA (2012). High serum sclerostin predicts the occurrence of osteoporotic fractures in postmenopausal women: the Center of Excellence for Osteoporosis Research Study. J Bone Mineral Res.

[14] Lee K (2020). Association of osteosarcopenic obesity and its components: osteoporosis, sarcopenia and obesity with insulin resistance. J Bone Mineral Metabolism.

[15] Seibel MJ (2005). Biochemical markers of bone turnover: part I: biochemistry and variability. Clin Biochem Rev.

[16] Shetty S, Kapoor N, Bondu JD, Thomas N, Paul TV (2016). Bone turnover markers: Emerging tool in the management of osteoporosis. Indian J Endocrinol Metabolism.

[17] Kuo T-R, Chen C-H (2017). Bone biomarker for the clinical assessment of osteoporosis: recent developments and future perspectives. Biomarker research.

[18] Greenblatt MB, Tsai JN, Wein MN (2017). Bone Turnover Markers in the Diagnosis and Monitoring of Metabolic Bone Disease. Clin Chem.

[19] Moriwaki K, Matsumoto H, Tanishima S, Tanimura C, Osaki M, Nagashima H, Hagino H (2019). Association of serum bone- and muscle-derived factors with age, sex, body composition, and physical function in community-dwelling middle-aged and elderly adults: a cross-sectional study. BMC Musculoskelet Disord.

[20] Vitale JA, Sansoni V, Faraldi M, Messina C, Verdelli C, Lombardi G, Corbetta S (2021). Circulating Carboxylated Osteocalcin Correlates With Skeletal Muscle Mass and Risk of Fall in Postmenopausal Osteoporotic Women. Front Endocrinol (Lausanne).

[21] Mera P, Laue K, Wei J, Berger JM, Karsenty G (2016). Osteocalcin is necessary and sufficient to maintain muscle mass in older mice. Mol Metab.

[22] Drey M, Sieber CC, Bertsch T, Bauer JM, Schmidmaier R (2016). Fi ATig: Osteosarcopenia is more than sarcopenia and osteopenia alone. Aging Clin Exp Res.

[23] Ostovar A, Nabipour I, Larijani B, Heshmat R, Darabi H, Vahdat K, Ravanipour M, Mehrdad N, Raeisi A, Heidari G (2015). Bushehr Elderly Health (BEH) Programme, phase I (cardiovascular system). BMJ open.

[24] Shafiee G, Ostovar A, Heshmat R, Darabi H, Sharifi F, Raeisi A, Mehrdad N, Shadman Z, Razi F, Amini MR (2017). Bushehr Elderly Health (BEH) programme: study protocol and design of musculoskeletal system and cognitive function (stage II). BMJ open.

[25] Aadahl M, Jorgensen T (2003). Validation of a new self-report instrument for measuring physical activity. Medicine and science in sports and exercise.

[26] Roberts HC, Denison HJ, Martin HJ, Patel HP, Syddall H, Cooper C, Sayer AA (2011). A review of the measurement of grip strength in clinical and epidemiological studies: towards a standardised approach. Age Ageing.

[27] Fried LP, Tangen CM, Walston J, Newman AB, Hirsch C, Gottdiener J, Seeman T, Tracy R, Kop WJ, Burke G (2001). Frailty in older adults: evidence for a phenotype. J Gerontol A Biol Sci Med Sci.

[28] Cruz-Jentoft AJ, Baeyens JP, Bauer JM, Boirie Y, Cederholm T, Landi F, Martin FC, Michel JP, Rolland Y, Schneider SM (2010). Sarcopenia: European consensus on definition and diagnosis: Report of the European Working Group on Sarcopenia in Older People. Age Ageing.

[29] Cruz-Jentoft AJ, Bahat G, Bauer J, Boirie Y, Bruyère O, Cederholm T, Cooper C, Landi F, Rolland Y, Sayer AA (2018). Sarcopenia: revised European consensus on definition and diagnosis. Age and ageing.

[30] Peck W: Consensus development conference: diagnosis, prophylaxis, and treatment of osteoporosis. Am J Med 1993, 94(6):646-650. 10.1016/0002-9343(93)90218-e 8506892

[31] Group WS (1994). Assessment of fracture risk and its application to screening for postmenopausal osteoporosis. Report of a WHO Study Group. World Health Organ Tech Rep Ser.

[32] Shafiee G, Ostovar A, Heshmat R, Keshtkar AA, Sharifi F, Shadman Z, Nabipour I, Soltani A, Larijani B: Appendicular Skeletal Muscle Mass Reference Values and the Peak Muscle Mass to Identify Sarcopenia among Iranian Healthy Population. International journal of preventive medicine 2018, 9. 10.4103/ijpvm.IJPVM_295_17 PMC5869961 29619149

[33] American Heart A, National Heart L, Blood I, Grundy SM, Cleeman JI, Daniels SR, Donato KA, Eckel RH, Franklin BA, Gordon DJ (2005). Diagnosis and management of the metabolic syndrome. An American Heart Association/National Heart, Lung, and Blood Institute Scientific Statement. Executive summary. Cardiol Rev.

[34] Hill KD, Farrier K, Russell M, Burton E (2017). Dysmobility syndrome: current perspectives. Clin Interventions Aging.

[35] Kirk B, Al Saedi A, Duque G (2019). Osteosarcopenia: A case of geroscience. Aging Med (Milton).

[36] Kumari C, Yagoub G, Ashfaque M, Jawed S, Hamid P (2021). Consequences of Diabetes Mellitus in Bone Health: Traditional Review. Cureus.

[37] Vs K, K P, Ramesh M, Venkatesan V (2013). The association of serum osteocalcin with the bone mineral density in post menopausal women. J Clin Diagn Res.

[38] Garnero P, Sornay-Rendu E, Duboeuf F, Delmas PD (1999). Markers of bone turnover predict postmenopausal forearm bone loss over 4 years: the OFELY study. J Bone Mineral Res.

[39] Muraca M, Cappariello A (2020). The role of Extracellular Vesicles (EVs) in the epigenetic regulation of bone metabolism and osteoporosis. Int J Molecular Sci.

[40] Gerdhem P, Ivaska KK, Alatalo SL, Halleen JM, Hellman J, Isaksson A, Pettersson K, Väänänen HK, Åkesson K, Obrant KJ (2004). Biochemical markers of bone metabolism and prediction of fracture in elderly women. J Bone Mineral Res.

[41] Neve A, Corrado A, Cantatore FP (2013). Osteocalcin: skeletal and extra-skeletal effects. Journal of cellular physiology.

[42] Baim S, Miller PD (2009). Assessing the clinical utility of serum CTX in postmenopausal osteoporosis and its use in predicting risk of osteonecrosis of the jaw. J Bone Miner Res.

[43] Wang M, Bolland M, Grey A (2016). Management recommendations for osteoporosis in clinical guidelines. Clin Endocrinol (Oxf).

[44] Tieland M, Trouwborst I, Clark BC (2018). Skeletal muscle performance and ageing. J Cachexia Sarcopenia Muscle.

[45] Terracciano C, Celi M, Lecce D, Baldi J, Rastelli E, Lena E, Massa R, Tarantino U (2013). Differential features of muscle fiber atrophy in osteoporosis and osteoarthritis. Osteoporos Int.

[46] Girgis CM, Cha KM, So B, Tsang M, Chen J, Houweling PJ, Schindeler A, Stokes R, Swarbrick MM, Evesson FJ (2019). Mice with myocyte deletion of vitamin D receptor have sarcopenia and impaired muscle function. J Cachexia Sarcopenia Muscle.

[47] Kim YM, Kim S, Won YJ, Kim SH (2019). Clinical manifestations and factors associated with osteosarcopenic obesity syndrome: a cross-sectional study in koreans with obesity. Calcified Tissue Int.

[48] Hovsepian S, Amini M, Aminorroaya A, Amini P, Iraj B (2011). Prevalence of vitamin D deficiency among adult population of Isfahan City, Iran. J Health Population Nutrition.

[49] Bruyère O, Cavalier E, Reginster J-Y (2017). Vitamin D and osteosarcopenia: an update from epidemiological studies. Curr Opinion Clin Nutrition Metabolic Care.

