# Supplementary material for: Association between biomarkers of bone health and osteosarcopenia among Iranian older people: The Bushehr Elderly Health (BEH) program
Source: BMC Geriatr. 2021 Nov 19;21:654. doi: 10.1186/s12877-021-02608-w (PMC8603566; doi:10.1186/s12877-021-02608-w)
Supplement: Supplementary file 1 — Additional file 1. [file 12877_2021_2608_MOESM1_ESM.docx]

**Supplement Table 1- normal range of bone biomarkers in this study**

|  | **Normal Range** | |
| --- | --- | --- |
| **Bone markers** | **Men** | **Women** |
| Osteocalcin, ng/ml | 24(14-46)* | 27(15-46)* |
| C-terminal cross-linked telopeptides (CTX) , ng/ml | 0.394±0.230** | 0.556±0.226** |
| Bone alkaline phosphatase(BALP), µg/L | 12.3±4.3** | 13.2±4.7** |
| Tartrate-resistant acid phosphatase (TRAP), U/L | 3.31±0.72** | 3.19±0.85** |
| Vitamin D, nmol/L | 75-250 | 75-250 |
| Calcium, mg/dl | 8.6-10.3 | 8.6-10.3 |
| Phosphorus , mg/dl | 2.6-4.5 | 2.6-4.5 |
| Alkaline phosphatase , U/L | 80-306 | 64-306 |

*median (Interquartile range)

**mean± SD

| **Supplement Table 2 - Comparison of bone markers between sub-groups in the study** | | | |  |
| --- | --- | --- | --- | --- |
| **Groups** | | | |  |
| **Bone marker** | **Osteosarcopenia/ Osteopenia/ osteoporosis (-)** | **Osteopenia/ osteoporosis (+)** | **Osteosarcopenia (+)** | **P-value** |
| Osteocalcin, ng/ml | 18.90(15.80-24.60) | 21.80(17.08-28.85) | 24.05(18.32-31.60) | 0.028 |
| C-terminal cross-linked telopeptides (CTX), ng/ml | 0.320 (0.230-0.540) | 0.400(0.310-0.575) | 0.465(0.340-0.598) | 0.009 |
| Bone alkaline phosphatase(BALP), µg/L | 13.70(11.40-16.60) | 16.30(13.08-20.60) | 16.29(13.20-20.90) | 0.018 |
| Tartrate-resistant acid phosphatase (TRAP), U/L | 2.90(2.50-3.50) | 3.40(2.80-4.10) | 3.65(3.20-4.30) | <0.001 |
| Vitamin D, nmol/l | 43.70(28.20-51.30) | 41.90(24.48-63.30) | 40.95(27.78-64.83) | 0.821 |
| Calcium, mg/dl | 9.40(9.00-9.70) | 9.20(9.00-9.60) | 9.30(9.10-9.50) | 0.403 |
| Phosphorus , mg/dl | 3.78(3.48-4.16) | 4.03(3.67-4.36) | 4.16(3.70-4.58) | 0.163 |
| Alkaline phosphatase , U/L | 188.0 (163.0-217.0) | 212.5(183.0-249.3) | 200.0 (169.0-237. 0) | 0.032 |
| Data are presented as median(Interquartile range) | | | |  |
